# Datasets of skills-rating questionnaires for advanced service design through expert knowledge elicitation

**DOI:** 10.1038/s41597-022-01421-3

**Published:** 2022-06-16

**Authors:** Hien Ngoc Nguyen, Ganix Lasa, Ion Iriarte, Ariane Atxa, Gorka Unamuno, Gurutz Galfarsoro

**Affiliations:** 1 Mondragon Unibertsitatea: Design Innovation Center (DBZ) - Faculty of Engineering Loramendi, 4, 20500 Arrasate - Mondragón, Gipuzkoa Spain; 2 IDEKO, Arriaga kalea, 2, E-20870 Elgoibar, Gipuzkoa Spain; 3 UROLA, Urola Kalea, s/n - Apdo 3, 20230 Legazpi, Gipuzkoa Spain

**Keywords:** Business, Industry

## Abstract

This article presents a dataset of service design skills which service design experts value as important requirements for design team members. Purposive sampling and a chain referral approach were used to recruit appropriate experts to conduct questionnaire-based research. Using the analytical hierarchy process (AHP), pairwise skills-rating questionnaires were designed to elicit the experts’ responses. The resulting dataset was processed using AHP algorithms programmed in R programming language. The transparent data and available codes of the research may be reused by design practitioners and researchers for replication and further analysis. This paper offers a reproduceable research process and associated dataset for conducting multiple-criteria decision analysis with expert purposive sampling.

## Abstract (summary)

**Table:** 

Measurement(s)	perspective of experts on important service design skills for design team members
Technology Type(s)	pairwise skills-rating questionnaires
Factor Type(s)	service design skills

## Background & Summary

Today, product-oriented companies are discovering new value creation methods that enable them to increase customer satisfaction, market share and competitiveness for improved economic returns and sustainability. New value creation can be achieved with new business models that help these companies to extend their services by means of their product-service systems (PSS), that is, systems representing bundles of products and services^1–3^. The existing literature often classifies these services according to three service groups: basic services (e.g., spare parts delivery and provision of tools and accessories), intermediate services (e.g., training, repair and maintenance), and advanced services^3–5^. In contrast to the first two classifications, advanced services offer new value creation by focusing on the delivery of product-service performance outcomes in terms of use-based and/or result-based contracts^4,6^. These contracts allow a customer to pay based on a result, output, performance and/or outcome of product-service delivery. Some typical cases of such contracts include the ‘power-by-the-hour’ model in terms of which Rolls-Royce receives a fixed price for each hour their engines work for customers^7^, and the ‘pay-per-lux’ model where the customer buys a subscription from Philips for a certain amount of light per year instead of buying Philips’ lamps^8^.

In order to design these advanced services, one of the key design elements is to equip the design team members (design practitioners) – or internal stakeholders of a company that seeks advanced service designs – with proper design skills (e.g., skills in market research or prototyping)^9^. This is important because design skills affect the key performance indicators in design work^4,10^ and help designers to understand their short-term functioning and long-term work development, enhancing the sustainable development of a company^11^. However, there are few research studies that identify which specific design skills are required by design teams^9,12^. To advance research in this area, a dataset was generated to answer the primary research question:

- *Who* (design team members, e.g., an engineer, a financial analyst, a marketer) needs to know and/or practice *what* design methods (e.g., interview techniques, prototyping) as design skills, to perform one or more design activities (e.g., to understand the customer’s latent needs, or to use wireframes for prototyping)?

The answer to this primary research question will also help design practitioners to build internal service capability (‘who needs to be trained in what’) and make decisions on training priorities in terms of their business resource constraints. Therefore, the captured dataset is also useful to answer the following two secondary research questions:

- *Who* should be trained in *what* design methods?
- *How* can these design methods be prioritized in building service capability (training and skills enhancement)?

The answers to the research questions can be varied, as they depend on the use context (e.g., company size, design knowledge and experience) and the perspective of the person answering the questions, leading to an unstructured decision problem. To tackle this problem, experts are in the best position to provide answers based on their expertise from both academic and industrial perspectives^13,14^. Therefore, the authors conducted an expert survey from which a dataset was developed to elicit expert knowledge related to the field of advanced service design in order to answer the research questions.

This dataset aims to enable design practitioners to determine which service design skills are valued for design teams from the perspective of service design experts, enabling practitioners to build internal service capability. Practitioners can use the dataset, methodology, data records and available R codes presented in the following sections to easily obtain expert knowledge for their own research contexts and practice. Researchers can also refer to this reproduceable research method for conducting multi-criteria decision analysis following expert purposive sampling.

## Methods

### Designing the expert survey

**Fig. 1:**
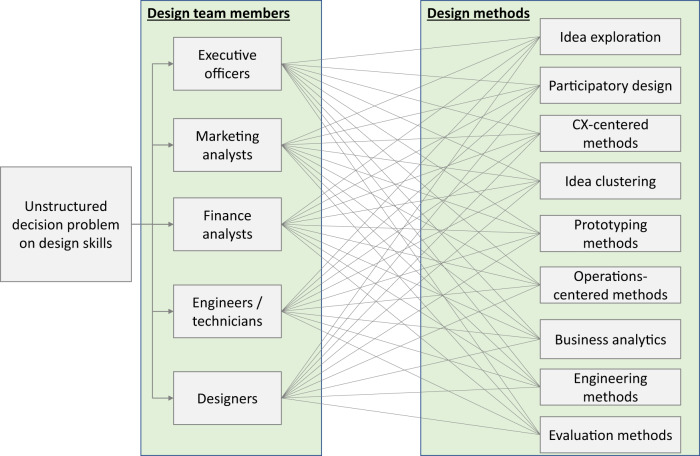
Unstructured decision problem on design skills. The decision problem is who (design team members) needs to know and/or practice what design methods, as design skills, to perform one or more design activities (e.g., to understand customer latent needs, to use the wireframes for prototyping). For further description of these design methods, refer to the dataset^21^ with the attached file name (.pdf): (Expert Survey) Skill-rating questionnaires.

The questionnaire design for the expert survey was based on the primary research question. In previous studies, researchers conducted a systematic review of the literature in the field of human-centered design for advanced services^12^ to define the two main elements of the primary research question: (1) who needs to know and/or practice (2) what design methods, as design skills, to perform one or more design activities. The systematic review resulted in: (1) five groups of design team members, and (2) nine groups of design methods, as summarized in Fig. 1. Figure 1 depicts an unstructured decision problem in which a design team member (e.g., an executive officer or a financial analyst) may employ one or more design methods (e.g., idea exploration or prototyping methods). The decisions can be varied, as they depend on the use context and the expertise of the person who makes the decision. As mentioned, the expertise of the service design experts was used to make these decisions as well as recommend to the design practitioners which decisions should be made.

To develop the right type of survey questionnaire, the authors applied the analytical hierarchy process (AHP) to design pre-coded (closed) pairwise questionnaires – based on a nine-point rating scale – for the expert survey. In the literature, the AHP is used to interrogate people who have extensive knowledge about a specific topic^15,16^; this method is commonly used for a small sample size^17^. It may also help experts or decision-makers to set priorities and make the best decision in a wide variety of decision situations in diverse fields, for example, design concept evaluation^16^, assessment of distribution center locations^18^, determination of potential groundwater recharge zones^19^, to name a few. The AHP has several functions, such as (i) breaking an unstructured problem down into rational hierarchical decision elements, and (ii) eliciting the best prioritized decisions from experts or decision-makers through questionnaires using pairwise comparisons of individual groups of elements. The answers to the survey provided by the experts can be varied, which would lead to inconsistency or subjective bias. This problem was avoided by validating the consistency of participants’ responses using consistency ratios (CRs) computed by the AHP^20^.

The authors broke down the primary research question by eliciting expert knowledge through pairwise skills-rating questionnaires, in accordance with the AHP. These skills-rating questionnaires of the expert survey are fully presented in the dataset^21^ with the attached file name (.pdf): *(Expert Survey) Skill-rating questionnaires*.

### Expert engagement

**Fig. 2:**
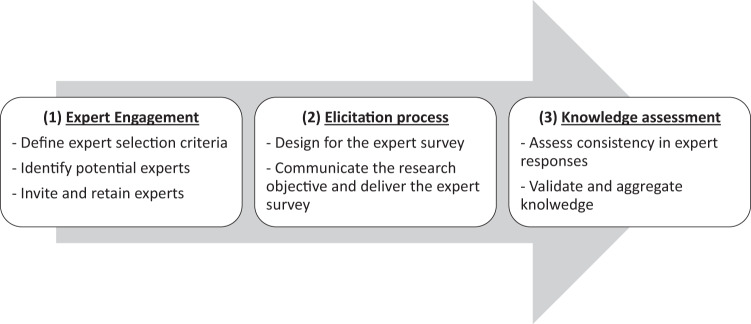
The procedure for the expert purposive sampling.

To effectively elicit expert knowledge on the primary research question using skills-rating questionnaires, a proper selection from the spectrum of experts was required. Therefore, the authors followed a rigorous sampling method, which is embraced by scientists as one of the purposive sampling techniques^22,23^. This sampling method, even more so with a small sample size, incorporates a measure of uncertainty in respect of the elicited expert knowledge and should therefore include an assessment of the validity of the findings^24^. This validity can be achieved by following the sampling procedure illustrated in Fig. 2.

Figure 2 starts with the expert engagement, in which the selection criteria for experts should be clearly defined^24–27^: (1) expertise relevant to the research question, (2) diversity in expertise, (3) willingness and dedicated to the research inquiry. Another expectation is related to the sample size of the expert panel. The literature suggests that the number of participants will vary according to the scope of the problem and the resources available (e.g., time and money)^28,29^. However, there is very little actual empirical evidence regarding the effect of the number of participants on the reliability or validity of consensus processes^30^. Because expert panels do not need to be representative samples for statistical purposes, representativeness is assessed based on the qualities of the expert panel following the expert selection criteria rather than the number of experts^31^. In practice, an empirical expert panel should consist of a minimum of 10 participants^16,32^.

Based on the expert selection criteria and the sampling guidance, the authors recruited 10 recognized experts, representing both industry and academia, from international workshops in the relevant fields; some of the experts were also selected using a chain referral approach in terms of which the initial experts nominated additional experts. These experts, whose profiles are presented in Table 1, have worked in various countries (the UK, France, Spain, Germany, and Japan), and represent diverse disciplines, such as human-centered design, related fields in Industry 4.0, servitization, business models and sustainable product-service systems. Therefore, the expert recruitment process ensured that their inputs were transdisciplinary.

**Table 1:** Expert profile.

Identification	Expertise	Major fields	Working years
Expert #1	Academist	Industrial engineering, Industry 4.0, servitization	33
Expert #2	Practitioner	Innovation and technology	29
Expert #3	Academist	Human-centered strategy for innovation, Industry 4.0	22
Expert #4	Practitioner	Research and development, innovation and servitization	20
Expert #5	Practitioner	Service engineering	19
Expert #6	Practitioner	Automation and digitalization in Industry 4.0, servitization	18
Expert #7	Academist	Sustainable product-service system, eco-innovation	14
Expert #8	Academist	Human-centered design, industrial design engineer	12
Expert #9	Practitioner	Digital manufacturing	10
Expert #10	Academist	Cyber physical systems, software engineering	7

### Elicitation process and knowledge assessment

After engaging the experts, the next step (see Fig. 2) was to send out the invitations and retain the experts via formal emails, which explained the topic of the research, namely design skills, and the research objectives. Next, the expert survey (the pdf file in the dataset^21^) was sent to the experts (see Table 1) via email in September 2021. All the expert responses were collected via returned emails around November 2021. The raw data (the expert responses) were inputted in the spreadsheet (the xlsx file in the dataset^21^). Lastly, the data were analyzed using the AHP with R codes (the html file in the dataset^21^), which resulted in the technical validation and aggregation of the experts’ answers to the primary and secondary research questions.

## Data Records

The presented dataset is stored at Mendeley Data (https://data.mendeley.com/, 10.17632/7brkgztjdx.3)^21^; the individual files are described below.

### (Expert Survey) Skill-rating questionnaires (.pdf)

This file presents the expert survey with the pairwise skills-rating questionnaires in accordance with the AHP. There are a total of nine skills-rating questionnaires – representing the nine groups of design methods – for the pairwise comparison of five groups of employees (the design team members). Each expert (see Table 1) answered each skill-rating questionnaire to evaluate to what extent a design method (e.g., idea exploration) is preferred by a job role (e.g., executive officers) compared to another job role (e.g., marketing analysts) using a nine-point rating scale.

### (Raw data) Skill-rating questionnaires through AHP (.xlsx)

This file contains the expert responses to the skills-rating questionnaires. The first column of the file sheet contains the design skills for rating, including nine groups of design skills that represent the nine skills-rating questionnaires. The second column indicates the pairwise comparison among the five groups of design team members for each skills-rating questionnaire. The next 10 columns display the raw responses of the experts, whose identifications are matched with those in Table 1, using the nine-point rating scale of the pairwise comparisons. The 13^th^ column stores the raw data in the form of CSV value strings used for their corresponding data inputs in R. The last column provides a summary of the data points and missing data points (NA): for a total of 862 data points, there are 38 missing datapoints (NA), that is, approximate 4.4% of the total data points.

### (R codes) AHP analysis and result (.html)

This file provides all the R codes^33^ for executing the AHP algorithms^34^ of the raw data (.xlsx). The missing data points (4.4% of the 862 data points) were also included without affecting the original dataset^35^. These R codes are presented in the four main sequenced sections: (i) R package preparation, (ii) data inputs, (iii) calculation of aggregated importance weights and (iv) calculation of the consistency ratios. The ‘R package preparation’ section presents the package instalment in the R environment to execute the AHP algorithms. The ‘Data inputs’ section indicates how the raw data (.xlsx) in the form of CSV value strings were inputted into R. The ‘Calculation of aggregated importance weights’ section indicates the aggregated results (see Table 2) of the expert decisions on the primary research question, namely ‘who needs to know and/or practice what design methods, as design skills, to perform one or more design activities’. This aggregated result was also used to answer the two secondary questions: (i) who should be trained in what design methods; and (ii) how can these design methods be prioritized in building service capability. Finally, the ‘Calculation of the consistency ratio’ section presents the validation results for the consistency of the expert responses.

**Table 2:** Aggregated importance weights and consistency ratio on each group of design methods with each group of design team members in accordance with AHP.

	Aggregated importance weights^a^					Total weight	Consistency ratio (CR)^a^
	Executive officers	Marketing analysts	Finance analysts	Engineers and/or technicians	Designers		
Idea exploration	0.133	0.170	0.063	**0.257**	**0.377**	1	0.16
Participatory design	0.099	**0.256**	0.069	**0.194**	**0.382**	1	0.10
CX-centered methods	0.079	**0.307**	0.064	0.183	**0.366**	1	0.08
Idea clustering	0.190	**0.274**	0.097	0.143	**0.296**	1	0.20
Prototyping methods	0.100	0.105	0.054	**0.308**	**0.434**	1	0.11
Operations-centered methods	0.169	0.120	0.074	**0.329**	**0.308**	1	0.12
Business analytics	**0.260**	0.172	**0.353**	0.090	0.125	1	0.13
Engineering methods	0.128	0.076	0.059	**0.501**	**0.237**	1	0.11
Evaluation methods	0.102	**0.282**	0.144	0.169	**0.303**	1	0.17

^a^For aggregated importance weights, the experts consistently indicated two to three groups of design team members—whose importance weight values are higher than 0.19, dominating that of the other groups in the total importance weight of 1—should acquire a corresponding group of design methods (skill sets). The values of CRs—that are not greater than 0.2—prove the responses of the experts on these questionnaires are tolerably consistent^17,36^. These CRs allow for the valid interpretation on the analysis result. The transparent data and available codes of the research are provided in the dataset^21^.

## Technical Validation

The answer to the primary research question depends on the expertise of the surveyed experts; the expert panel did not need to be a representative sample for statistical inferences^30–32^. Therefore, the qualities of the expert panel, based on the expert selection criteria, were more critical for the analytical validity of this dataset than the number of participants. Moreover, the application of the AHP method to data analysis does not require a large sample size for statistical validity^17^; however, the expert responses represent subjective judgement based on the experts’ expertise. Therefore, the consistency ratios had to be calculated to justify the consistency of the expert responses.

Based on the mathematical algorithms of the AHP^34^, programed for its computation in the language of R^33^, Table 2 summarizes the results of the analysis of the expert responses (the html file in the dataset^21^), including the consistency ratios and aggregated importance weights. The former indicates that all the values of the consistency ratios are not greater than 0.2, proving that the aggregated responses of the experts on these questionnaires are tolerably consistent^17,36^. This means that the interpretation of the aggregated importance weights is technically valid. The aggregated importance weights indicate that a group of design team members (e.g., executive officers or designers) needs to know and/or practice a group of design methods (e.g., idea exploration or prototyping methods) to a greater extent than other groups of design team members, with a total importance weight of 1. These aggregated importance weights reveal the answers to the primary and secondary research questions, which are further discussed in the next section.

Despite the rigorousness of this research, the authors acknowledge that there are limitations associated with pre-coded (closed) skills-rating questionnaires. These closed questionnaires in practice do not allow for other possible choices (design team members and design methods); this limitation of closed-ended questionnaires has also been acknowledged by other questionnaire-based research studies^16,37,38^. For instance, the expert or design practitioner may consider the role of the sales team in addition to the defined design team members (see Fig. 1) for advanced service designs. Therefore, the study findings need to be adapted to specific business contexts. Nevertheless, the validity of the expert responses was assessed to guarantee the technical validity of the analysis results, and an acceptable level of judgement bias was ensured based on the consistency ratios, as discussed above.

## Usage Notes

To replicate this research, researchers and design practitioners should follow the procedures presented in the Methods section. Based on the research context, the content of the expert survey, which consisted of skills-rating questionnaires, and the expert selection criteria should be adopted. The methodology for collecting and analyzing datasets should follow the instructions documented in the Data Records section. The analysis of datasets can easily be accomplished reusing the R codes for the AHP algorithms (see the Code Availability section).

Researchers and design practitioners may reuse the analysis results of this research study’s dataset (see Table 2) to look for practical applications by answering the research questions. First of all, for Table 2 the consistency ratios should not be greater than 0.2; if they are, the researchers should improve the survey design to ensure an acceptable level of consistency in the expert responses before further analysis. Subsequently, the aggregated importance weights indicate that the experts consistently indicated two to three groups of design team members – whose importance weight values are higher than 0.19, dominating those of the other groups in the total importance weight of 1 – need to know and/or practice a corresponding group of design methods (skill sets).

For the primary research question, for example in the skill set of ‘idea exploration’, the ‘designers’ and ‘engineers and/or technicians’ – whose importance weights are 0.257 and 0.377, respectively, in the total importance weight of 1 (see Table 2) – preferably need to master the skill set better than the other groups of design team members in terms of the aggregated perspectives of all surveyed experts. Based on these aggregated importance weights, the same reasoning is applicable to the rest of the design team members and groups of design methods.

Similarly, the answers to the two secondary research questions – (i) who should be trained in what design methods, and (ii) how can these design methods be prioritized in building service capability – are also based on the aggregated importance weights. For instance, in the skill set of ‘participatory design’, the ‘designers’, ‘marketing analysts’, and ‘engineers and/or technicians’ – who have the highest aggregated importance weights of 0.382, 0.256, and 0.194, respectively, in order – should be prioritized for the training of the skill set in the same order. As can be seen in Table 2, the skills of ‘designers’ are in the highest demand, except for the skill set of ‘business analytics’ (e.g., game theory, profit formula), which should be represented to a greater extent by ‘executive officers’ and ‘financial analysts’. In addition to designers, ‘engineers’ should not only be competent in technical skills (‘prototyping methods’, ‘operations-centered methods’ and ‘engineering methods’). They should preferably be trained to know the skill sets of ‘idea exploration’ and ‘participatory design’ used to understand both the tangible and latent requirements of customers.

In summary, the dataset and its analysis results enable researchers and design practitioners to build a transdisciplinary design team in which each group of design methods can be handled by two or three job roles, in the order of priority.

## Data Availability

The code availability for open access is given by the dataset^21^: *(R codes) AHP analysis and result.html*. These codes are written in R language (version 4.1.2, https://r-project.org) to input the raw data (.xlsx), run the AHP algorithm and produce the final result summarized in Table 2. For further description of the R codes, refer to the section Data Records.
